# Early Animal Origin of BACE1 APP/Aβ Proteolytic Function

**DOI:** 10.3390/biology13050320

**Published:** 2024-05-04

**Authors:** James A. Langeland, Lillian Baumann, Eva M. DeYoung, Raphaela Angelina Varella, Nkatha Mwenda, Alejandro Aguirre, D. Blaine Moore

**Affiliations:** Department of Biology, Kalamazoo College, 1200 Academy Street, Kalamazoo, MI 49006, USA

**Keywords:** β-amyloid (Aβ), BACE, evolution, amyloid precursor protein (APP), animal

## Abstract

**Simple Summary:**

One feature of Alzheimer’s disease is the accumulation β-amyloid (Aβ) in the brain. Since the BACE1 protease is required to produce Aβ, and is a potential clinical target, it is of interest to know when its enzymatic function evolved and for what purpose. Here, we show that BACE1 likely evolved from a gene duplication event near the base of the animal clade, well before the evolution of the APP/Aβ substrate.

**Abstract:**

Alzheimer’s disease is characterized, in part, by the accumulation of β-amyloid (Aβ) in the brain. Aβ is produced via the proteolysis of APP by BACE1 and γ-secretase. Since BACE1 is the rate-limiting enzyme in the production of Aβ, and a target for therapeutics, it is of interest to know when its proteolytic function evolved and for what purpose. Here, we take a functional evolutionary approach to show that BACE1 likely evolved from a gene duplication event near the base of the animal clade and that BACE1 APP/Aβ proteolytic function evolved during early animal diversification, hundreds of millions of years before the evolution of the APP/Aβ substrate. Our examination of BACE1 APP/Aβ proteolytic function includes cnidarians, ctenophores, and choanoflagellates. The most basal BACE1 ortholog is found in cnidarians, while ctenophores, placozoa, and choanoflagellates have genes equally orthologous to BACE1 and BACE2. BACE1 from a cnidarian (*Hydra*) can cleave APP to release Aβ, pushing back the date of the origin of its function to near the origin of animals. We tested more divergent BACE1/2 genes from a ctenophore (*Mnemiopsis*) and a choanoflagellate (*Monosiga*), and neither has this activity. These findings indicate that the specific proteolytic function of BACE1 evolved during the very earliest diversification of animals, most likely after a gene-duplication event.

## 1. Introduction

Alzheimer’s disease (AD) is a devastating neurodegenerative disorder that results in progressive changes in cognition, behavior, and mood [1]. Many proteins are implicated, including Tau, but the precipitating event in AD appears to be the abnormal buildup of extracellular plaques in the brain containing β-amyloid (Aβ) [1]. Aβ, a 4 kDa peptide, is derived from the amyloid precursor protein (APP) via proteolysis [2]. APP is expressed in all human tissues, with the APP695 isoform being most commonly expressed in neurons [3]. APP is proteolyzed by β-secretase, generating a c-terminal fragment with 99 amino acids (C99), which is then cut by γ-secretase to produce Aβ, which is secreted into the extracellular space [3].

The β-secretase involved in the cleavage of APP to produce Aβ has been identified as BACE1 [4]. The BACE1 homologue BACE2 has been found to be irrelevant to Aβ production [5]. BACE1 is the primary β-secretase in the brain and the rate-limiting enzyme in the production of Aβ, as previous studies have shown Aβ secretion to be inhibited in BACE1 knockout mice [6]. However, BACE1 knockout studies have shown that BACE1 may have some essential functions. For example, BACE1 knockout mice were found to have delayed myelination and thinner myelin in central and in peripheral nerves [7]. Additionally, mice lacking BACE1 have abnormal hippocampal synaptic plasticity and cognitive performance [8], and BACE1 knockout mice had decreased grip strength and increased sensitivity to pain [7]. As BACE1 may be a target for AD treatments, it is necessary to understand the essential functions that would be impacted by such a treatment. Given the undesirable side effects associated with BACE1 inhibitors such as Verubecestat and Lanabecestat [9,10], it is of interest to more fully understand the implications of BACE1 inhibition. Knowing precisely when BACE1 evolved, and for what purpose, will be important context for eventual therapeutic inhibition.

We have previously shown that BACE1 and Aβ evolved asynchronously [11]. Phylogenetic analysis showed that BACE1 arose near the origin of animals, while the Aβ substrate evolved hundreds of millions of years later and cannot be found outside of vertebrates. Furthermore, functional analysis showed that BACE1 from an animal (the cephalochordate *Branchiostoma floridae*) that never evolved Aβ can nonetheless proteolyze the APP/Aβ substrate. These findings indicate that BACE1 has deeply conserved and essential functions that have nothing to do with APP/Aβ processing per se, but that were later co-opted into this processing. 

In the current study, we perform a functional evolutionary analysis of BACE1 APP/Aβ proteolysis in taxa flanking the base of the animal clade. Animal origins remain opaque, and early animal phylogeny is unresolved; while choanoflagellates are accepted as a sister group of animals, the relationships of the non-bilaterian basal animal groups of porifera, ctenophores, and cnidaria are not agreed upon [12,13,14,15]. Our aim is not to resolve these relationships, but rather to probe where we can find BACE1 orthologs in these taxa and to determine whether they can proteolyze APP/Aβ. Through our functional analysis, we find that cnidaria are the most basal taxon to have a bona fide BACE1 ortholog, and through our functional assay we find that this ortholog can proteolyze human APP to release Aβ.

## 2. Materials and Methods

### 2.1. Phylogenetic Analysis

Our previously described search approach [11] remained focused on basal animals and yielded new sequences from cnidarians (*Orbicella faveolata* XP_020602753.1, *Nematostella vectensis* EDO39359.1, *Actinia tenebrosa* XP_031568918.1, *Stylophora pistillata* XP_022793028.1 and *Pocillopora damicornis* XP_027053766.1), placozoans (*Trichoplax adhaerens* RDD46120; RDD46118.1), ctenophores (*Pleurobrachia bachei* Neurobase sb|2666892|), sponges (*Amphimedon queenslandica* XP_003385244.1, *Oopsacas minuta* KAI6660898.1, *Aphrocallistes vastus* CAC83293.1, *Halisarca dujardinii* QSX72298.1), and choanoflagellates (*Salpingoeca rosetta* XP_004993482.1). We were also able to add to our previously identified *Mnemiopsis* partial transcript (ML154145a) by fusing it with an overlapping partial sequence ML1541_cuf_154 (Mnemiopsis Genome Project Portal). Multiple sequence alignments and evolutionary history inferences were produced using MEGA 11 [16]. The evolutionary history was inferred by using the Maximum likelihood method and JTT matrix-based model [17], and a bootstrap consensus tree was inferred from 100 replicates [18]. Branches corresponding to partitions reproduced in less than 50% of bootstrap replicates were collapsed. Initial tree(s) for the heuristic search were obtained automatically by applying Neighbor-Joining and BioNJ algorithms to a matrix of pairwise distances estimated using the JTT model, and then selecting the topology with the superior log likelihood value. A discrete Gamma distribution was used to model evolutionary-rate differences among sites (5 categories (+G, parameter = 2.1022)). For purposes of clarity, the final tree produced excludes some non-informative sequences from our cnidarian, sponge, and ctenophore sampling. Tests for positive selection around BACE active sites were carried out using the codon-aligned BACE nucleotide files in the Datamonkey implementation of HyPhy. All sequences, alignments, and tree files are available as Supplementary Materials.

### 2.2. Expression Constructs

For each relevant BACE amino acid sequence (*Hydra*, *Mnemiopsis*, and *Monosiga*—see [11], for sequence IDs), full-length cDNAs were synthesized with codon optimization for CHO cells (GenScript, Piscataway, NJ, USA). These cDNAs were cloned into pcDNA3.1(+) expression plasmids with either C-HA or C-GFP tags, and industrial-grade plasmid preps were obtained (GenScript). *Branchiostoma floridae* BACE1 construct detail is provided in [11]. 

### 2.3. Cell Culture and Transfection

Chinese Hamster Ovary cells stably transfected with the 695 amino acid variety of human APP (CHO 695) were maintained in minimum essential alpha media (αMEM; Invitrogen, Carlsbad, CA, USA) supplemented with 10% fetal bovine serum, 1% glutamine, and 1% penicillin/streptomycin (Invitrogen). The cells were passaged as specified in Balan et al. [19]. The cells were seeded into 6-well plates at a density of 1.25 × 10^4^ prior to transient transfection. CHO 695 cells at 70% confluence were transfected with GenePorter (Genlantis, San Diego, CA, USA) 24 h after plating, following manufacturer’s instructions. In total, 2 μg DNA (GFP, *Homo* BACE1, *Branchiostoma* BACE1, *Hydra* BACE1, *Mnemiopsis* BACE 1/2 or *Monosiga* BACE 1/2) and 10 μL transfection reagent were delivered in 1.0 mL serum-free αMEM for five hours. Transfections were stopped by the addition of 1.0 mL serum-containing αMEM. The visual inspection of GFP controls verified successful transfection. Successful localizations of BACE constructs were confirmed with GFP-tagged versions of each BACE construct. Microscopy was performed on an inverted Nikon DIAPHOT 200 compound light microscope (Melville, NY, USA). 

### 2.4. Collection of Conditioned Media and ELISA

Conditioned media were harvested 16 h following transfection. A complete Mini protease-inhibitor cocktail (Roche, Indianapolis, IN, USA) was used to prevent the degradation of secreted proteins. In total, 1.0 mL of media was transferred to microfuge tubes containing protease inhibitors and incubated on ice. The tubes were centrifuged at 13,000 RPM for 20 min at 4 °C. The supernatant was transferred to new microfuge tubes and stored at −80 °C until analysis. Secreted Aβ 1–40 was measured via a human-specific ELISA kit (Invitrogen) following the manufacturer’s instructions. The absorbance was measured at 450 nm on a μquant microplate spectrophotometer (Biotek, Winooski, VT, USA). The data were normalized to total protein levels measured in cell lysates by the BCA protein assay (Thermo Fisher Scientific, Waltham, MA, USA). See details on lysate preparation and BCA protein assay in Balan et al. [19]. 

### 2.5. Statistics

The significance of ELISA data was determined by a one-way analysis of variance (ANOVA) followed by Tukey’s post hoc testing.

## 3. Results

### 3.1. BACE Phylogenetic Analysis

**Figure 1:**
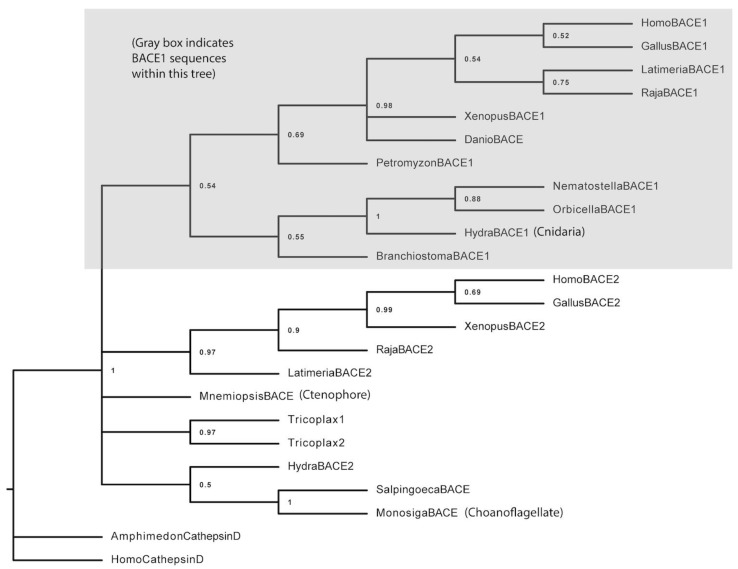
**Cnidarians are the most basal group with a BACE1 ortholog.** Maximum-likelihood tree of BACE sequences with nodes collapsed below the 50% bootstrap support. BACE1 and BACE2 sequences form clear clades with cnidarians being the most basal group found in our search to have a BACE1 sequence (shown in the gray box). Other BACE-like sequences can be found in basal animal taxa such as ctenophores (e.g., *Mnemiopsis*) and placozoans (*Tricoplax*), as well as in the sister group to animals, choanoflagellates (*Monosiga*). These sequences form a polytomy with the BACE1 and BACE2 groups, mirroring the polytomies that are found in phylogenies at the whole-organism level. As a tentative model, we consider them to be single-gene, pre-duplicate precursors to BACE 1 and BACE 2. *Tricoplax* appears to have undergone an independent duplication of this BACE1/2 precursor. Cathepsin D aspartyl proteases are the sister group to the BACE genes; we can find this gene but not BACE genes in porifera (*Amphimedon*).

We searched genomic databases of basal animal species for BACE1 orthologs. Our phylogenetic analysis (Figure 1) shows that cnidaria (e.g. *Hydra vulgaris*) have a bona fide BACE1 ortholog (see the gray box). They are the most basal group to have this gene, albeit weakly supported with a 54% bootstrap value. We also find clear BACE orthologs in ctenophores (e.g., *Mnemiopsis leidyi*) and placozoans (*Trichoplax adhaerens*), as well as in choanoflagellates (e.g *Monosiga bevicollis* and *Salpingoeca rosetta*). Together with a divergent *Hydra* BACE (termed BACE2 in the database), these more basal BACE genes form a polytomy with the BACE1 and BACE2 clades. Sponges (e.g., *Amphimedon* in our tree) do not appear to have BACE 1 genes; the closest sequence is a Cathepsin D, a related aspartyl protease. A simple but highly tentative model to explain our tree would be that the BACE1 and BACE 2 gene families arose via gene duplication in the cnidarian+bilaterian ancestor. We were unable to detect signals of positive selection in or around BACE active sites that may have resulted from this duplication. Given that the phylogeny of the basal animal taxa themselves are unresolved, it is not surprising that the base of our BACE tree is unresolved. What is clear is that cnidaria are the most basal group to have a bona fide BACE1 gene.

### 3.2. Analysis of BACE Functional Activity 

**Figure 2:**
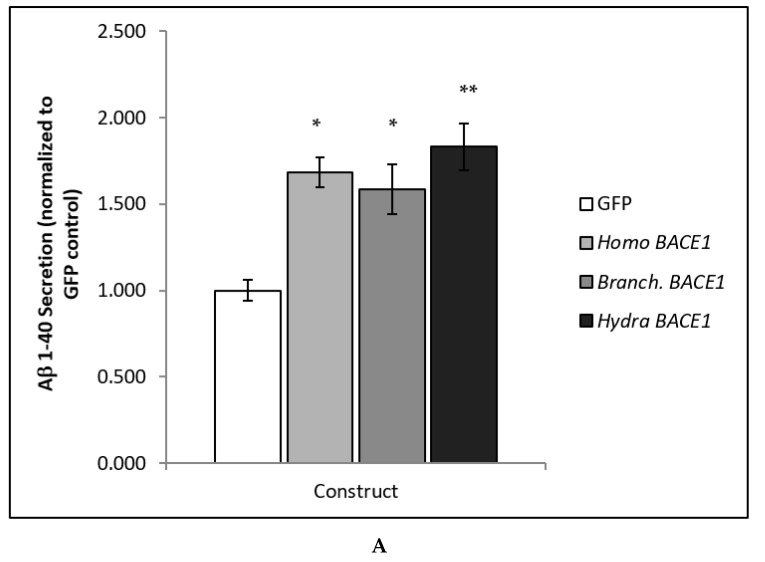

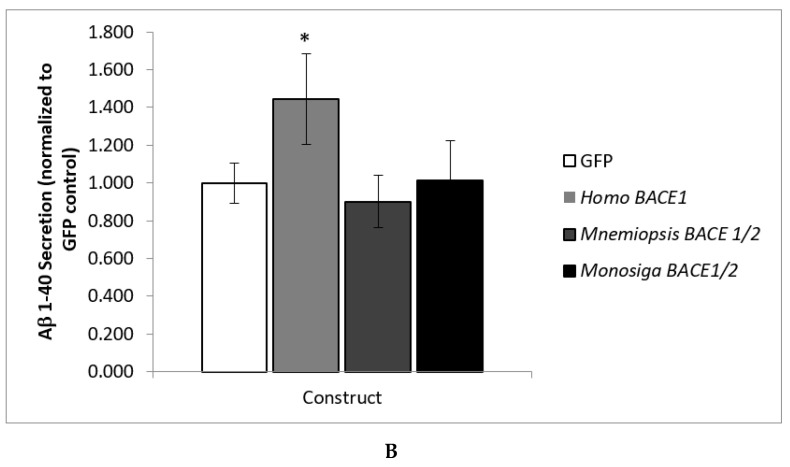
**BACE1 functional activity towards human APP/Aβ is conserved in cnidarians but not in ctenophores or choanoflagellates**. CHO 695 cells stably transfected with human APP were transiently transfected with the following cDNA expression constructs: GFP (negative control), *Hydra* BACE1, *Mnemiopsis* BACE 1/2, or *Monosiga* BACE 1/2. *Homo* BACE1 and *Branchiostoma* (*Branch.*) BACE1 constructs were used as positive controls. Conditioned media were harvested after 16 h, and the secretion of human Aβ was determined via ELISA. Data were normalized to total protein and analyzed with one-way ANOVA followed by Tukey’s post hoc test (* *p* < 0.05; ** *p* < 0.01). Data are representative of three independent transfection rounds. *Homo*, *Branchiostoma*, and *Hydra* BACE1 all elevated Aβ secretion (**A**), while *Mnemiopsis* and *Monosiga* BACE 1/2 did not (**B**).

The functional activity of BACE proteins was examined in our well-described in vitro model system, the CHO695 cell line, which stably expresses the 695-amino acid, a primarily neuronal isoform of human APP (CHO695 cells; [11]). Using this system, we previously showed that BACE1 functional activity towards APP/Aβ extends to the cephalochordate subphylum [11]. To determine whether the BACE1 proteolysis of human APP is conserved in cnidarians, CHO695 cells were transfected with *Hydra* BACE1. GFP transection was used as a negative control, while *Homo* BACE1 was used as a positive control. BACE1 from the cephalochordate *Branchiostoma* (*Branch.*), which we have previously shown to be a functional ortholog of *Homo* BACE1, was transfected for comparison and served as a positive control. Sixteen hours after transfection, conditioned media were collected and analyzed with a 1–40 Aβ sandwich ELISA. A bicinchoninic acid (BCA) protein assay was performed on cell lysate samples and used to normalize Aβ levels and account for any minor variation in the total protein or cell number. A one-way ANOVA revealed significant differences in Aβ secretion (Figure 2A) [F(3,32) = 5.2398, *p* < 0.01]. Tukey’s post hoc testing showed a significant increase in Aβ secretion for both *Homo* BACE1 and *Branchiostoma* BACE1 (*p* < 0.05), and a significant increase in *Hydra* BACE1 (*p* < 0.01), confirming our phylogenetic analysis and proving cnidarian BACE1 is a functional ortholog of human BACE1. 

Next, we turned our attention to ctenophores and choanoflagellates to determine if more divergent BACE genes show functional activity towards APP/Aβ. We transfected CHO695 cells with *Mnemiopsis* BACE 1/2 or *Monosiga* BACE 1/2, again using GFP and *Homo* BACE1 as negative and positive controls. We note that despite exhaustive searches, we were not able to obtain full-length sequences of either gene. Our *Mnemiopsis* sequence does encompass the known full-length mature BACE1 (BACE1 is proteolyzed prior to function) [4], but our *Monosiga* sequence is missing one of two known BACE1 active sites. Aβ concentration in conditioned media samples was determined via an Aβ 1-40 ELISA, and total protein levels from cell lysate samples were used to normalize secretion data. A one-way ANOVA revealed significant differences in Aβ secretion when comparing GFP, *Homo* BACE1, *Mnemiopsis* BACE 1/2 (Figure 2B), and *Monosiga* BACE 1/2 [F(3,45) = 4.0945; *p* < 0.05]. Tukey’s post hoc analysis showed that *Homo* BACE1 significantly elevated Aβ secretion (*p* < 0.05), while GFP control, *Mnemiopsis* BACE 1/2, and *Monosiga* BACE 1/2 did not. We confirmed successful transfection and sub-cellular localization via fluorescence microscopy using GFP-tagged versions of *Mnemiopsis* and *Monosiga* BACE 1/2 proteins (see Supplemental Figure S1), indicating that a lack of Aβ secretion is not due to a failure of expression or proper localization. Our results demonstrate that BACE proteolysis towards APP/Aβ is not conserved in the divergent BACE1/2 gene present in ctenophores, reinforcing the conclusion that it is BACE1-specific and found only in the bilateria+cnidaria group. Our result with *Monosiga* is consistent with this conclusion but must be taken with greater caution given that we know one of two active sites is lacking. For completeness, future studies can include full-length choanoflagellate BACE 1/2 sequences and placozoa BACE1/2 sequences. 

## 4. Discussion

**Figure 3:**
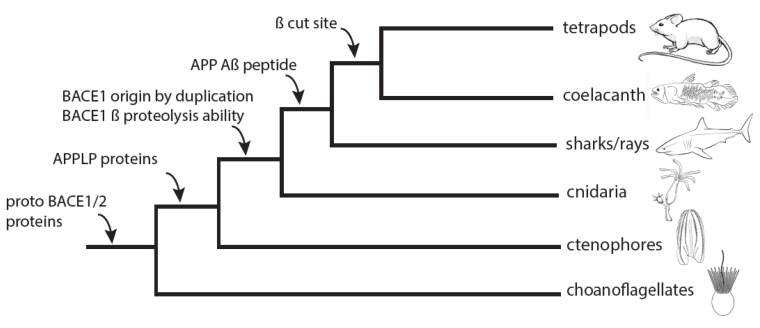
**Relative timeline for BACE1 and Aβ evolution**. Simplified animal phylogeny with key events supported in this paper and our previous paper [11]. Pre-duplicate BACE1/2 predates animal origins and can be found in extant choanoflagellates, as well as in ctenophores and placozoans (not shown for simplicity). Cnidaria are the most basal group that definitively shows BACE1. We demonstrate that this gene can proteolyze APP/Aβ, while BACE1/2 genes cannot. This proteolytic ability thus correlates with the likely advent of BACE1 by gene duplication. Strikingly, the ability of BACE1 to proteolyze APP/Aβ predates the actual origin of Aβ in vertebrates by several hundred million years.

The present study documents when BACE orthologs evolved the ability to proteolyze APP/Aβ. Our earlier work demonstrated that APP-like proteins are found throughout most animal taxa, but sequences homologous to Aβ only evolved within gnathostomes (jawed vertebrates), and the β cut site is only conserved within sarcopterygians (lobe-finned fishes). Further, we demonstrated the functional conservation of the BACE1 proteolysis of APP/Aβ in a cephalochordate (e.g., *Branchiostoma floridae*). These new data, combining phylogenetic and functional analyses, push the experimentally verified proteolysis further back to cnidarians (e.g., *Hydra vulgaris*). Hydra never evolved the Aβ sequence, and their evolutionary history predates the origin of Aβ by hundreds of millions of years. Our demonstration that BACE1 from *Branchiostoma* and *Hydra*, which never evolved the Aβ sequence, can proteolyze human APP and liberate Aβ indicates a very high level of functional conservation since the origin of BACE1 and suggests BACE1 has deeply conserved and essential functions that have nothing to do with APP processing. The fact that BACE1/2 from ctenophores and choanoflagellates is unable to proteolyze human APP indicates that functional BACE activity towards APP/Aβ is unique to the BACE1 clade. Indeed, cnidaria are the most basal group to have BACE1, and the most basal group to have a BACE gene capable of cleaving APP. While the base of our tree remains unresolved, the combination of phylogenetic with functional analyses strongly suggests that this specific proteolytic activity evolved in the ancestor of cnidarians+bilterians, likely after a gene duplication (see Figure 3 for a summary). 

Our data re-emphasize that BACE1 has deeply conserved and essential functions that are independent of APP/Aβ proteolysis. BACE1 has multiple substrates besides APP, and some of its potential non-amyloidogenic roles are beginning to emerge. Among the more intriguing ones are the BACE1 proteolysis of NRG1 during myelination [20], the cleavage of SEZ6 and the promotion of dendritic branching [21], and the proteolysis of CHL1, resulting in semaphorin-regulated growth cone collapse [22]. These essential developmental functions likely represent the ancestral role of BACE1 in animals, but further work will be needed to demonstrate this.

Comparisons between BACE1 and BACE2 show that the two homologous sequences are distinct in function and regulation. BACE2 is expressed at low levels in the brain but is highly expressed in peripheral tissues such as in the pancreas, colon, and kidney [23]. BACE2 functions include regulating glucose homeostasis in beta-islet cells and pigmentation in melanosomes [24]. Furthermore, BACE2 does not appear to proteolyze APP to produce Aβ; instead, it cleaves within the Aβ sequence at the theta cut site [25] and lowers Aβ secretion from cultured cells [26]. 

Alzheimer’s disease is the fifth-leading cause of death among Americans 65 and older and costs billions of dollars annually [27,28]. The development of therapeutics is critically important, and BACE1 inhibition remains a preferred target for AD therapeutics despite setbacks and adverse side effects [9,10]. Further elucidation of the basal animal species in which BACE proteolytic function arose and its core biological functions and substrates will help inform treatment strategies and their implications. In particular, it may prove necessary to develop BACE modulators that selectively inhibit the beta-cleavage of APP. It will also be of interest to determine the functional origin of the APP β-cleavage site. Aβ-containing APPs extend deep into vertebrate phylogeny, but not beyond vertebrates [11]. Elucidating which taxa retain bona fide BACE1 substrates will help complete the picture of BACE1 evolution vis-a-vis APP/Aβ proteolysis and will further contextualize our finding that BACE1 proteolytic function evolved during the very earliest diversification of animals. 

## 5. Conclusions

In conclusion, we have performed a detailed phylogenetic and functional biochemical examination of the evolutionary history of BACE1, the rate-limiting protease responsible for liberating the Aβ peptide from amyloid precursor protein in Alzheimer’s disease. Using a combination of phylogenetic and functional cell-based biochemical analyses, we have demonstrated that the most basal BACE1 ortholog is found in cnidarians, while ctenophores and choanoflagellates have genes equally orthologous to BACE1 and BACE2. A cnidarian BACE1 (*Hydra vulgaris*) can cleave human APP to release Aβ, but neither ctenophore nor choanoflagellate BACE1/2 has this activity. These findings indicate that the proteolytic function of BACE1 evolved deep in the animal lineage, likely after a gene-duplication event.

## Data Availability

The original contributions presented in the study are included in the article/Supplementary Material, further inquiries can be directed to the corresponding author/s.

## Supplementary Materials

The following supporting information can be downloaded at https://www.mdpi.com/article/10.3390/biology13050320/s1: Figure S1: Successful expression and similar localization of BACE proteins.
