# Supplementary material for: Early Animal Origin of BACE1 APP/Aβ Proteolytic Function
Source: Biology (Basel). 2024 May 4;13(5):320. doi: 10.3390/biology13050320 (PMC11117577; doi:10.3390/biology13050320)
Supplement: Supplementary file 1 [file biology-13-00320-s001.zip › biology-2928354-supplementary.pdf]

A.

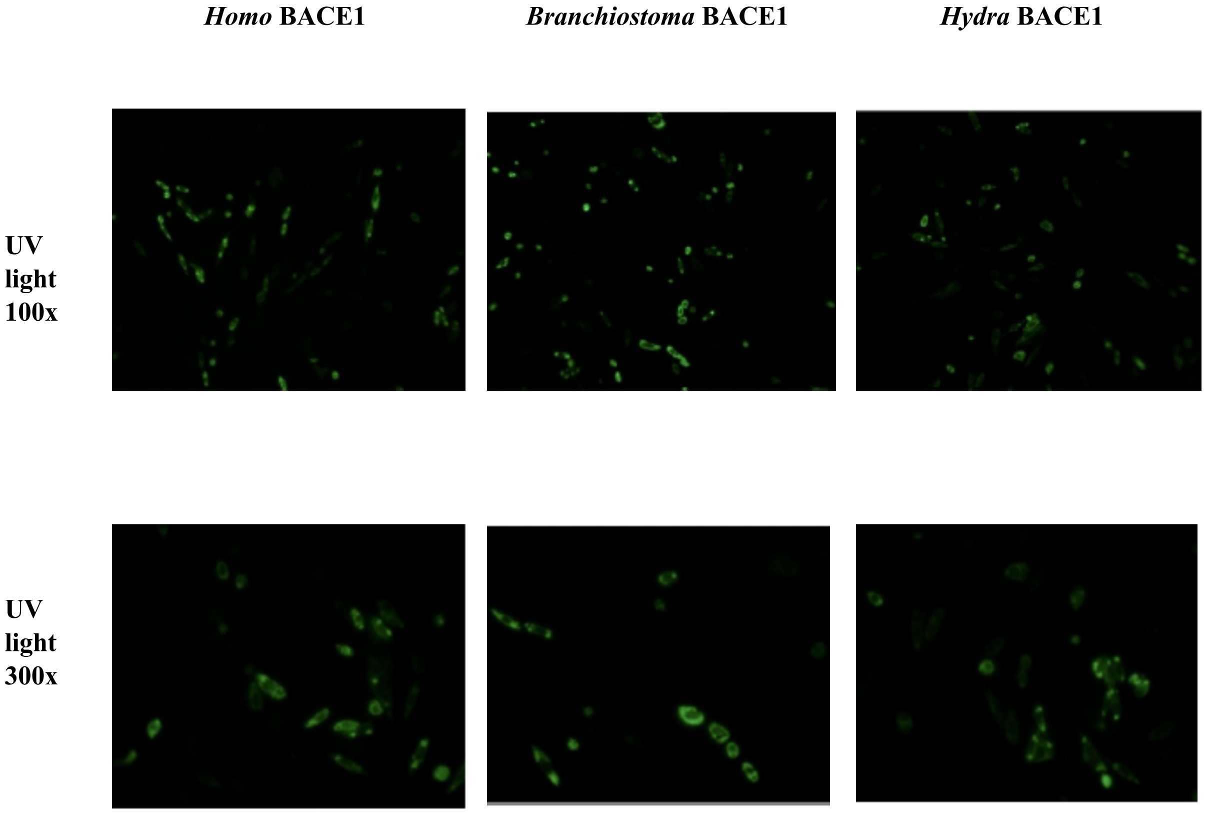
Homo BACE1
Branchiostoma BACE1
Hydra BACE1
UV light 100x
UV light 300x

B.

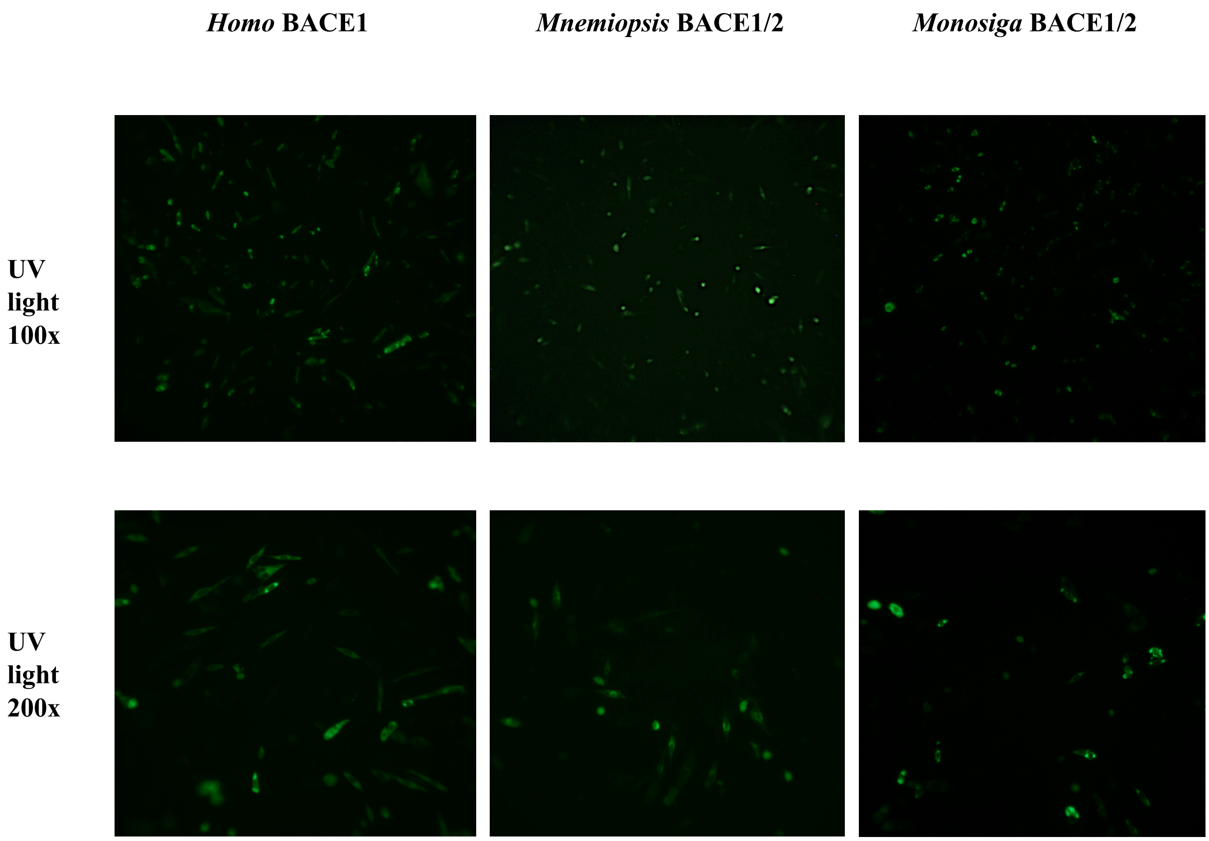

Supplemental Figure S1: Successful expression and similar localization of BACE proteins. CHO695 cells were transfected with 2.0 µg of green florescent protein (GFP)-tagged BACE using 10 µL of genePORTER (GP). Images were taken under UV light at 100x, 200x or 300x magnification. No differences in BACE subcellular localization were noted for any species. Therefore, lack of functional activity for *Mnemiopsis* BACE1/2 and *Monosiga* BACE1/2 is not due to altered expression or localization.
